# Tuberculosis Case Fatality and Other Causes of Death among Multidrug-Resistant Tuberculosis Patients in a High HIV Prevalence Setting, 2000-2008, South Africa

**DOI:** 10.1371/journal.pone.0144249

**Published:** 2016-03-07

**Authors:** Martie van der Walt, Joey Lancaster, Karen Shean

**Affiliations:** Tuberculosis Platform, South African Medical Research Council, Pretoria, South Africa; University of Pittsburgh Center for Vaccine Research, UNITED STATES

## Abstract

**Introduction:**

South Africa has the highest reported rates of multi-drug resistant TB in Africa, typified by poor treatment outcomes, attributable mainly to high default and death rates. Concomitant HIV has become the strongest predictor of death among MDR-TB patients, while anti-retroviral therapy (ART) has dramatically reduced mortality. TB Case fatality rate (CFR) is an indicator that specifically reports on deaths due to TB.

**Aim:**

The aim of this paper was to investigate causes of death amongst MDR-TB patients, the contribution of conditions other than TB to deaths, and to determine if causes differ between HIV-uninfected patients, HIV-infected patients receiving ART and those without ART.

**Methods:**

We carried out a retrospective review of data captured from the register of the MDR-TB programme of the North West Province, South Africa. We included 671 patients treated between 2000–2008; 59% of the cohort was HIV-infected and 33% had received ART during MDR treatment. The register contained data on treatment outcomes and causes of death.

**Results:**

Treatment outcomes between HIV-uninfected cases, HIV-infected cases receiving ART and HIV-infected without ART differed significantly (p<0.000). The cohort death rate was 24%, 13% for HIV-uninfected cases and 31% for HIV-infected cases. TB caused most of the deaths, resulting in a cohort CFR of 15%, 9% for HIV-uninfected cases and 20% for HIV-infected cases. Cohort mortality rate due to other conditions was 2%. AIDS-conditions rather than TB caused significantly more deaths among HIV-infected cases receiving ART than those not (p = 0.02).

**Conclusions:**

The deaths among HIV-infected individuals contribute substantially to the high death rate. ART co-therapy protected HIV-infected cases from death due to TB and AIDS-conditions. Mechanisms need to be in place to ensure that HIV-infected individuals are retained in care upon completion of their MDR-TB treatment.

## Introduction

Globally there were an estimated 250 000 cases of multi-drug resistant (MDR) tuberculosis (TB) in 2009[1] with South Africa (SA) having the highest reported rates in Africa. MDR-TB is typified by poor treatment outcomes, [2] mainly attributable to high default and death rates. Concomitant HIV has become the strongest predictor of death among MDR-TB patients, although anti-retroviral therapy (ART) has dramatically reduced mortality in these patients.

Even though it is recognised that TB disease itself is responsible for a large proportion of deaths among patients treated for TB, data on the relative contribution of TB as compared to other causes is unknown. Information on TB as a specific cause of death (TB specific case fatality) for a country can only be gleaned on a population-wide level from death notification records, but as such cannot be linked to specific TB cohorts or sub-groups treated during a specific year. The indicator of TB Case Fatality is seldom reported on its own for surveillance and TB impact measurements.[3,4] This said, this TB-specific case fatality rate (CFR) is an important epidemiological indicator and a measure to directly determine the impact of new interventions, such as diagnostics or new drug regimens, on survival of TB patients. [5,6]

We were particularly interested in deaths due directly to TB, and the relative contribution of AIDS-related sequelae on the additional deaths among the HIV-infected cases. Little is known about the specific mechanisms of continued high deaths rates of HIV-patients on ART [7] while few studies report on long-term survival of MDR-TB/HIV patients. [8,9,10] While some publications on case fatality for fully sensitive TB can be found, [11–16] there is less literature available on those of MDR-TB. [7,9,10] It is expected, as for susceptible TB, that MDR-TB CFR be much lower than the overall TB death rate, especially in high HIV burden settings. Deaths due to TB and HIV-related sequelae are largely preventable due to currently available diagnostic and treatment options although many of these deaths can still be attributable to health system failures. The MDR-TB programme in the North West Province (NWP), one of the nine provinces in SA, keeps a register in which the specific cause of death including AIDS-related sequelae, is recorded for each patient.

The aim of this study was to investigate causes of death amongst a large cohort of MDR-TB patients, the relative contribution of conditions other than TB to death, and to determine how causes of death differ among HIV-infected patients depending on the provision and timing of ART. In addition, we used data on post-treatment deaths to determine the long-term survival of the cohort.

## Methods

We conducted a retrospective review of data routinely collected in a MDR-TB register kept by the NWP (this being the only province to keep a register at this time), consisting of data on causes of death, HIV status and ART usage. We included all patients with culture-confirmed MDR-TB treated between 2000–2008. Causes of death data were as per verbal autopsy.

### HIV and Anti-retroviral treatment programme in the NWP

From 2003 all TB patients in NWP had access to HIV Voluntary Counselling and Testing (VCT) and were eligible for ART when CD4 cell count <450. Since 2006 ART eligibility criteria w changed to offer ART to MDR-TB patients regardless of CD4 cell count. Patients not yet on ART at onset of MDR treatment were not automatically initiated on ART, due to concerns around pill burden, overlapping toxicities and IRIS. ART initiation was usually delayed for four months (or at the end of the intensive phase).

### Data Analysis

The TB-specific CFR was calculated as the number of deaths specifically due to the TB disease, divided by the cohort size. This differed from the death rate which is the overall number of all deaths of patients that died during treatment divided by the cohort size. The sum of the CFR and a mortality rate calculated from deaths due to other causes equals the death rate. Data elements captured in the register were: HIV-coinfection, ART usage, and treatment outcomes. Patients who had received no ART during treatment and who were referred for ART once MDR-TB treatment had been completed were defined in our analyses as “No ART”. Patients already on ART at MDR-TB treatment initiation were defined as “ART during treatment”. A small number of patients were started on ART at the end of the intensive phase of treatment, and included in the latter group. For treatment outcome analysis we combined patients with unknown outcome with default. The categories still on treatment and treatment stopped due to side effects with failure.

### MDR-TB treatment

The NWP started treating MDR-TB patients under DOTS-Plus [17] in 2000, described elsewhere in more detail. [18] This consisted of a standardised regimen with an intensive phase of four months with five drugs, and a continuation phase, of three drugs for a minimum of 18 months. From 2005/6 the guidelines changed [19] and the intensive phase was changed to at least six months with a total duration of treatment for a minimum of 24 months. All patients were admitted to the in-patient facility for initiation of MDR-TB treatment and discharged when sputum culture negative.

### Post-treatment follow-up

Patients returned to the hospital at six-monthly intervals for wellness care. Post-treatment follow-up was censored at the end of 2010. The post-treatment follow-up period ranged between two years (patients who started in 2008) and ten years for patients who started in 2000. Patients were asked about recurrent TB, and if available the sensitivity pattern was acquired. Patients not attending the pre-scheduled follow up visit were fastidiously traced, and if found to have died, cause of death information was collected where available. Since 2008 the province has used an electronic DR-Register for DR-surveillance.

### Ethics

This was a retrospective review of information previously collected in the provincial registers during routine practise. No patient identifiers were entered in the register; data were abstracted by patient record number.

## Results

### Treatment outcome and causes of death

**Fig 1:**
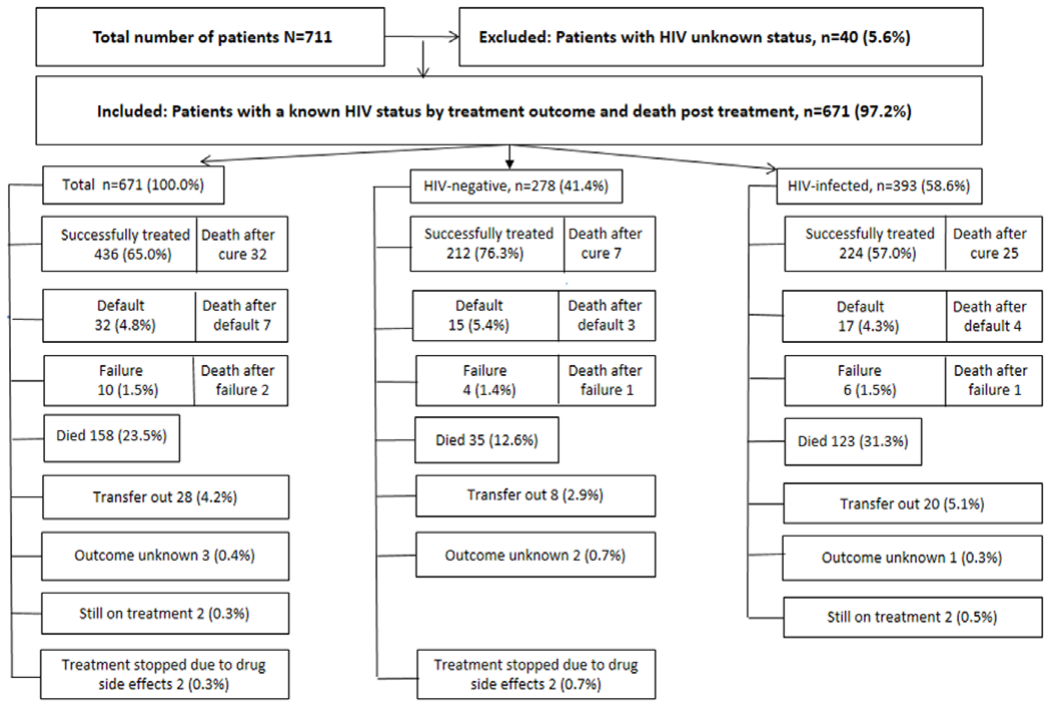
Treatment outcomes and HIV infection among multidrug resistant tuberculosis patients in the North West Province, South Africa, 2000–2008, N = 711.

Between 2002–2008 711 MDR-TB patients were treated at the NWP MDR-TB hospital, of which 671 had a known HIV result, and of whom 58.6% (393/671) tested HIV-infected (Fig 1). In this reduced cohort of 671 patients, 65.0% (436/671) were successfully treated (defined as treatment cure plus treatment completion), 4.8% (32) defaulted, 1.5% (10) failed treatment and 23.5% (158) died (Fig 1). The death rate among the HIV-infected group was almost double (31.3%) compared to those of the HIV-uninfected group 12.6% (35/278) while more of the latter group were successfully treated (76.3%) vs. 57.0% of the HIV-infected. Treatment outcomes differed significantly among HIV-uninfected cases, HIV-infected cases receiving ART during treatment and HIV-infected cases not on ART (p<0.001, 95% CI, data not shown).

### TB Specific Case Fatality Rates

In this cohort of 671 patients 158 deaths occurred during treatment (Fig 1 and Table 1), with only 101 specifically caused by the TB infection, resulting in a 15.1% (101/671) cohort TB-specific CFR. The CFR for HIV-uninfected patients was 8.6%; 24/278 and for HIV-infected patients 19.6% (77/393). The cohort mortality rate due to other causes was 1.8% and the surplus deaths due to HIV was 6.7% (Table 1). Among the HIV-uninfected patients we found that 11 of the deaths were not attributable to TB, but due to other conditions; while among the HIV-infected cases other conditions caused fewer deaths (0.8%). The other conditions were corpulmonale, diabetes, pneumonia, liver disease and epilepsy. Two patients died from non-pathological reasons (viz. a motor vehicle accident and suicide; Table 1).

**Table 1:** Causes of death among multi-drug resistant tuberculosis patients who died during treatment or follow-up, North West Province, South Africa, 2000–2008, N = 199.

Cause of death and treatment stage	HIV Infection Status								Total	
	HIV-infected and access to ART						HIV-negative			
	ART during treatment		No ART		Total					
	n	%	n	%	n	%	n	%	n	%
**Death during treatment**										
TB	27	79.4	50	56.2	77^3^	62.6	24^3^	68.6	**101**^3^	**63.9**
AIDS^1^	7	20.6	38	42.7	45	36.6	0	-	**45**^4^	**28.5**
Other^2^	0	-	1	1.1	1	0.8	11	31.4	**12**^5^	**7.6**
Total	34	100.0	89	100.0	123	100.0	35	100.0	**158**^6^	**100.0**
**Death post treatment**										
TB	1	50.0	11	39.3	12	40.0	5	45.5	**17**	**41.5**
							0	-	**17**	**41.5**
							6	54.5	**7**	**17.1**
AIDS^1^	1	50.0	16	57.1	17	56.7				
Other^2^	0	-	1	3.6	1	3.3				
Total	2	100.0	28	100.0	30	100.0	11	100.0	**41**	**100.0**
**Overall Total**	**36**		**117**		**153**		**46**		**199**	

^1^ = AIDS-related sequelae: liver failure, cancer, *Cryptococcus* meningitis, lactic acidosis, Kaposi sarcoma, malaria, *Pneumocystis* pneumonia and a pulmonary embolus

^2^ = Other causes: liver disease, corpulmonale, diabetes, pneumonia, epilepsy, motor vehicle accident and suicide

^3^ = Cohort TB specific case fatality rate = 101/671 = 15.1%; Rate among HIV-negative = 24/278 = 8.6%; Rate among HIV-positive = 77/393 = 19.6%

^4^ = Cohort Rate of mortality due to other causes = 12/671 = 1.8%

^5^ = Cohort Surplus deaths due to HIV-infected patients = 45/671 = 6.7%

^6^ = Cohort Death rate = 158/671 = 23.5%

### Use of ART among HIV-infected MDR-TB patients

Although 393 individuals were HIV-infected and thus eligible for ART (Fig 1) only 92/393 had already had access to ART previously, with an additional three starting during treatment. Treatment outcomes between the “ART during treatment” group and the “No ART” patients were similar (p = 0.22; 95% CI; Table 2). However causes of death differed significantly between the two ART groups (p = 0.02, 95% CI; Table 1): TB as the main cause among patients who received ART during treatment, whereas among the “No ART” group deaths were equally attributable to TB and AIDS. The AIDS-related sequelae were liver failure, cancer, *Cryptococcus* meningitis, lactic acidosis, Kaposi sarcoma, malaria, *Pneumocystis* pneumonia and a pulmonary embolus in one patient. No deaths due to IRIS occurred.

**Table 2:** Treatment outcomes among HIV-infected multi-drug resistant tuberculosis patients and access to ART, North West Province South Africa, 2000–2008, N = 393.

Treatment Outcomes*	Access to ART				Total	
	ART during treatment		No ART			
	n	%	n	%	n	%
Died	34	35.8	89	29.9	123	31.3
Successfully treated	52	54.7	172	57.7	224	57.0
Default	6	6.3	12	4.0	18	4.6
Transfer out	1	1.1	19	6.4	20	5.1
Failure	2	2.1	6	2.0	8	2.0
**Total**	**95**	**100.0**	**298**	**100.0**	**393**	**100.0**

*P = 0.22, 95% CI

### Long-term post-treatment outcomes

An additional 41 patients died during the post-treatment follow-up period (Fig 1 and Table 1), thereby increasing the long term number of deaths in this cohort to 199 and the long term cohort death rate to 30.1%. Overall, 17/41 (41.4%) deaths were due to recurrent TB. Of the eleven deaths among HIV-uninfected patients, TB and other conditions caused half each of the deaths. Under the HIV-infected patients, 28/30 deaths occurred among those who had not received any ART during treatment (Table 1). Of the 11/12 of the HIV-infected patients who were diagnosed with recurrent TB fell within the “No ART” group.

## Discussion

This study on the causes of death among MDR-TB patients spans a period of nine years in a setting with a high TB/HIV-coinfection burden. The MDR-TB programme in the NWP of SA ran a MDR-TB programme with close follow up of patients while on MDR-TB treatment as well as post treatment, and tracing of patients if visits were not attended. During the study period the overall successful treatment rate was 65%, which are higher than the average rate in South Africa or globally. [2] As such the WHO target of 75% for successful treatment rates for MDR-TB is not an unrealistically high target if high death and default rates can be addressed. [1] The successful outcome rate in our study is comparable to that achieved in other MDR-TB treatment programmes, with largely HIV-uninfected patients, in other parts of the world, [20–22] while our high death rate is similar to that found in other high co-infection settings. [22,23] Despite our high death rate we demonstrate that high successful treatment rates can be achieved in high burden TB/HIV settings, but require intensive strategies to prevent treatment default.

Globally there is a paucity of data looking at causes of death among MDR-TB patients, irrespective of HIV status, both during and after treatment. This excellent data on causes of death enabled us to calculate TB-specific case fatality rates. Accurate estimates of TB specific mortality are crucial for the proper evaluation of TB control programmes. Whereas the overall death rate was 24%, the TB-specific case fatality rate was much lower at 16%. As expected, the case fatality rate was lower for the HIV-uninfected sub-group (9%), but still higher than a reported rate of 5% for drug sensitive TB [16]. Fifty five percent of the MDR-TB patients in our cohort were HIV-infected, and we were also able to quantify the relative contribution of AIDS-sequelae, other than TB, as cause of death. Among HIV-infected cases AIDS-sequelae was the major cause of death, as expected, while for patients HIV-uninfected the majority of deaths were due to TB. Overall, other pathological conditions and non-diseases contributed very little to the overall death rates. The long term death rate of the cohort increased to 29.6%, whereas almost two thirds fell under the HIV-infected patient sub group, thus making HIV-infected patients significantly more likely to die post-treatment than their HIV-uninfected counterparts (P = 0.015, 95% CI). However, almost all of these post treatment deaths were from the No ART patients. We had no information on whether the No ART patients had indeed accessed HIV treatment and care after completion of their MDR treatment, but our data shows that these patients remained at higher risk of death than their counterparts who had ART.

The quality of the evidence on the impact of ART among HIV-infected MDR-TB patients is weak, [26,27] as is information to clarify the mechanisms behind the high death rate and on the optimal time to start ART.[1,28] The decision on when to start ART should be no different than that for a susceptible TB patient, i.e. as early as possible and once TB treatment is being tolerated, and at least within the first two months.[28,29] In our cohort few patients received any ART during treatment (25%). Despite the expected protection from death for patients who received ART during treatment, as suggested by Palacois [30] we could not confirm this as the death rates were comparable (36% and 30% respectively for ART during treatment and No ART groups). However, when taking the post treatment deaths into account, we confirmed the increased of survival for HIV-coinfected MDR-TB patients using ART.

The main limitations of this study are related to the retrospective nature of the data collected under routine TB control programme settings. There was no CD4 cell count information in the register, which results would have enabled us to further investigate the association between CD4 cell count and death. Furthermore we had no data on time to death or on time from diagnosis to treatment start which would have allowed us to stratify our analysis by patients who died early on in treatment vs. those who died later on in treatment. We also had no access to patient demographic and other clinical data, which could have explained some of our findings. Similarly, genotyping of the MDR isolate and the recurrent isolate was not done, which may have provided information on relapse or a new infection. Generalisation of our findings were compounded by the fact that there was considerable variation between other cohort sizes and settings. [20,22,24,25] However, our large sample size provided reliable data on the relative proportion of deaths due to TB in a high HIV-prevalence cohort, as well as the surplus deaths due to HIV. Due to the paucity of clinical data we were also unable to look for significant differences with regards to age, income etc. between the HIV infected and uninfected groups.

Future research should aim to identify patients at risk of clinical deterioration as early as possible during treatment and to intervene appropriately to prevent death. Furthermore, the mechanisms behind patients dying early on in treatment opposed to those who die later on in treatment need to be elucidated, as it is unknown if severe disease at onset of treatment leads to early death as compared to patients who die later on during treatment. Furthermore, due to recurrent TB and its impact on long term death rates, the optimal duration of follow up after MDR treatment need to be determined, and how this follow up can be done in the most cost effective manner.

Findings on death after successful outcome also needs in depth exploration and related recommendations for prevention strategies devised.
